# Meta-analysis on the efficacy of allogeneic hematopoietic stem cell transplantation to treat malignant lymphoma

**DOI:** 10.1515/biol-2022-0771

**Published:** 2024-05-31

**Authors:** Jin Zhao, Xiaojing Guo, Meijing Zheng, Liping Su

**Affiliations:** The Department of Hematology, Shanxi Provincial Cancer Hospital, Taiyuan, 030013, Shanxi, China; Hematology Department of Shanxi Hospital, Cancer Hospital, Chinese Academy of Medical Sciences, Taiyuan, 030013, Shanxi, China; Department of Hematology, Affiliated Cancer Hospital, Shanxi Medical University, Taiyuan, 030013, Shanxi, China

**Keywords:** autologous hematopoietic stem cell transplantation, allogeneic hematopoietic stem cell transplantation, malignant lymphoma, meta-analysis

## Abstract

The goal of the study involved the comparison of clinical efficacy of allogeneic hematopoietic stem cell transplantation (allo-HSCT) and autologous hematopoietic stem cell transplantation (auto-HSCT) in the treatment of malignant lymphoma (ML). The effectiveness of allo-HSCT versus auto-HSCT in the treatment of ML was compared by searching EMBASE, PubMed, Web of Science, and the Cochrane Library for relevant studies. The confidence intervals (CI) and odds ratio (OR) of the article’s outcomes were described by a forest plot. Finally, 972 patients in seven articles were included. Overall survival (OS) did not differ significantly between allo-HSCT and auto-HSCT groups (OR  =  0.87, 95% CI: 0.66–1.14, *P*  =  0.31). Furthermore, there was no significant difference in adverse reactions (AR) between the two groups (OR  =  1.35, 95% CI: 0.81–2.24, *P*  =  0.25). We observed a significant difference in progression-free survival (PFS) between the two groups (OR  =  4.14, 95% CI: 2.93–5.35, *P* < 0.01). There was no evidence of publication bias in this meta-analysis. The incidence of OS and AR differ significantly between allo-HSCT and auto-HSCT, but the PFS was longer in ML patients who received allo-HSCT.

## 1 Introduction

Malignant lymphoma (ML) is a group of malignant tumors originating from lympho-hematopoietic tissue. ML is a broad term describing malignant tumors that arise from lymph nodes or lymphoid tissues in other organs. It is further subdivided into Hodgkin’s lymphoma (HL) and non-Hodgkin’s lymphoma (NHL) [1,2]. Its clinical manifestations are lymph node enlargement, accompanied by fever, rash, emaciation, and anemia, accounting for about 5% of all malignant tumors [3], ranking 11th–13th in the incidence of tumors. The incidence of lymph cancer in China is increasing at an annual growth rate of 3–5%, which has a younger trend, and the incidence of patients aged 30–40 is also increasing [4].

The diagnosis of ML mainly depends on pathological and immunological examinations of tumor tissues, which brings many difficulties for clinicians to obtain pathological specimens and diagnosis, so it is easy to cause misdiagnosis and missed diagnosis, thus delaying the best treatment time [5]. As a vital means to strive for long-term survival and cure of hematological malignancies, allogeneic hematopoietic stem cell transplantation (allo-HSCT) has unique advantages in the treatment of hematological malignancies as a treatment method that utilizes hematopoietic stem cells of donors to reconstruct the hematopoietic and immune systems of patients. It can cure over 70 diseases including leukemia, severe aplastic anemia, and inherited metabolic disorders [6,7]. Survival advantages have been observed in patients treated with autologous hematopoietic stem cell transplantation (auto-HSCT) for extremely aggressive and relapsed/refractory lymphoma [8]. In recent years, with the continuous advancement of studies on HSCT, the clinical outcome of this treatment has made significant progress [9]. Allo-HSCT can select the appropriate preparative regimen and donors for transplantation according to the individual differences of patients to reduce the recurrence after transplantation.

Combining allo-HSCT with targeted therapy and immunotherapy enhances the clinical efficacy of ML patients [10,11]. This meta-analysis aims to systematically estimate the clinical efficacy of auto-HSCT and allo-HSCT to treat ML, thereby providing more evidence-based medical evidence for treating such patients.

## 2 Materials and methods

### 2.1 Inclusion and exclusion criteria

#### 2.1.1 RCT studies in English

Study subjects: (1) Patients were diagnosed with ML using pathology, bone marrow cell morphology, and immunohistochemistry. (2) Patients who underwent a comprehensive examination before transplantation and had no severe underlying disease or infection met the indications of allo-HSCT treatment.

Exclusion criteria: (1) patients with other malignant tumors, (2) patients with mental or organic diseases, and (3) ongoing study or the study that had unpublished or unspecified results during the literature search.

### 2.2 Literature screening and data extraction

The data were extracted and cross-examined by two researchers independently, and then evaluated by a third researcher. The extracted contents included basic information (author, publication date), study objects, intervention measures, and clinical outcome indicators (progression-free survival [PFS], AR, overall survival [OS], cumulative incidence of relapse [CIR], and non-relapse mortality [NRM]).

### 2.3 Literature retrieval

From their respective inceptions until April 2023, PubMed, EMBASE, the Web of Science, and the Cochrane Library were searched. The search terms included HSCT, stem cell transplantation or hematopoietic, transplantation or hematopoietic stem cell, lymphoma, lymphomas, sarcoma or germinoblastic, germinoblastic sarcoma, sarcomas or germinoblastic, reticulolymphosarcomas, reticulolymphosarcoma, germinoblastoma, lymphoma or malignant and ML, allo-HSCT, transplantation of allogeneic hematopoietic stem cells, allogeneic transplantation, ML, and lymphoma.

### 2.4 Study outcomes

Outcomes of interests were PFS, adverse reactions, OS, CIR, and NRM. Eligibility criteria for inclusion in this meta-analysis were not limited to the application of specific definitions.

### 2.5 Risk assessment of literature bias

Two reviewers appraised the risk of bias in the included studies independently and cross-checked the results. The risk of bias was evaluated using Cochrane-recommended risk of bias in RCT evaluation instruments [12].

### 2.6 Statistical analysis

RevMan5.4 statistical software was used for data analysis. The odds ratio (OR) with a 95% confidence interval (CI) was used to assess the effect. The included literature was analyzed through a heterogeneity test, and *P* value and *I* ^2^ were adopted to analyze the heterogeneity quantitatively. Data were mapped by forest plots to evaluate the consequences. The funnel plot was applied to evaluate publication bias.

## 3 Results

### 3.1 Included studies

**Figure 1:**
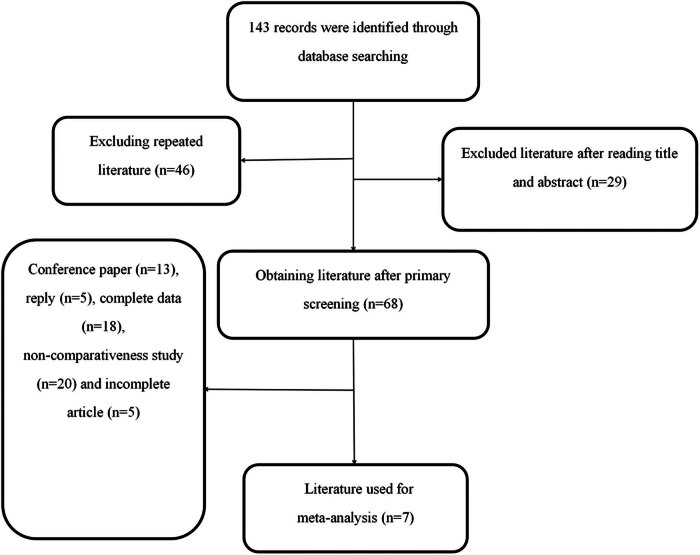
Flow chart of the literature retrieval and study collection.

Total of 143 articles were identified from the databases and manual retrieval. After exploring the title and abstract, 29 studies were excluded, and 46 studies were excluded due to repeated reports. Seven studies were included after excluding 61 (13 conference papers, 5 replies, 18 complete data, 20 non-comparativeness studies, and 5 incomplete articles). Figure 1 depicts the procedure of literature evaluation.

### 3.2 Basic characteristics and evaluation result of the risk of bias of included literature

**Figure 2:**
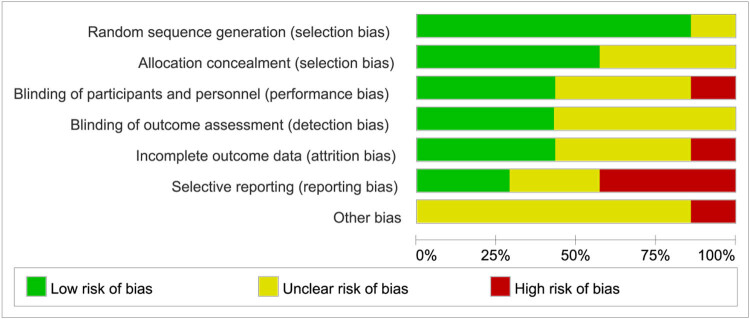
Summary of risk assessment of included literature.

**Figure 3:**
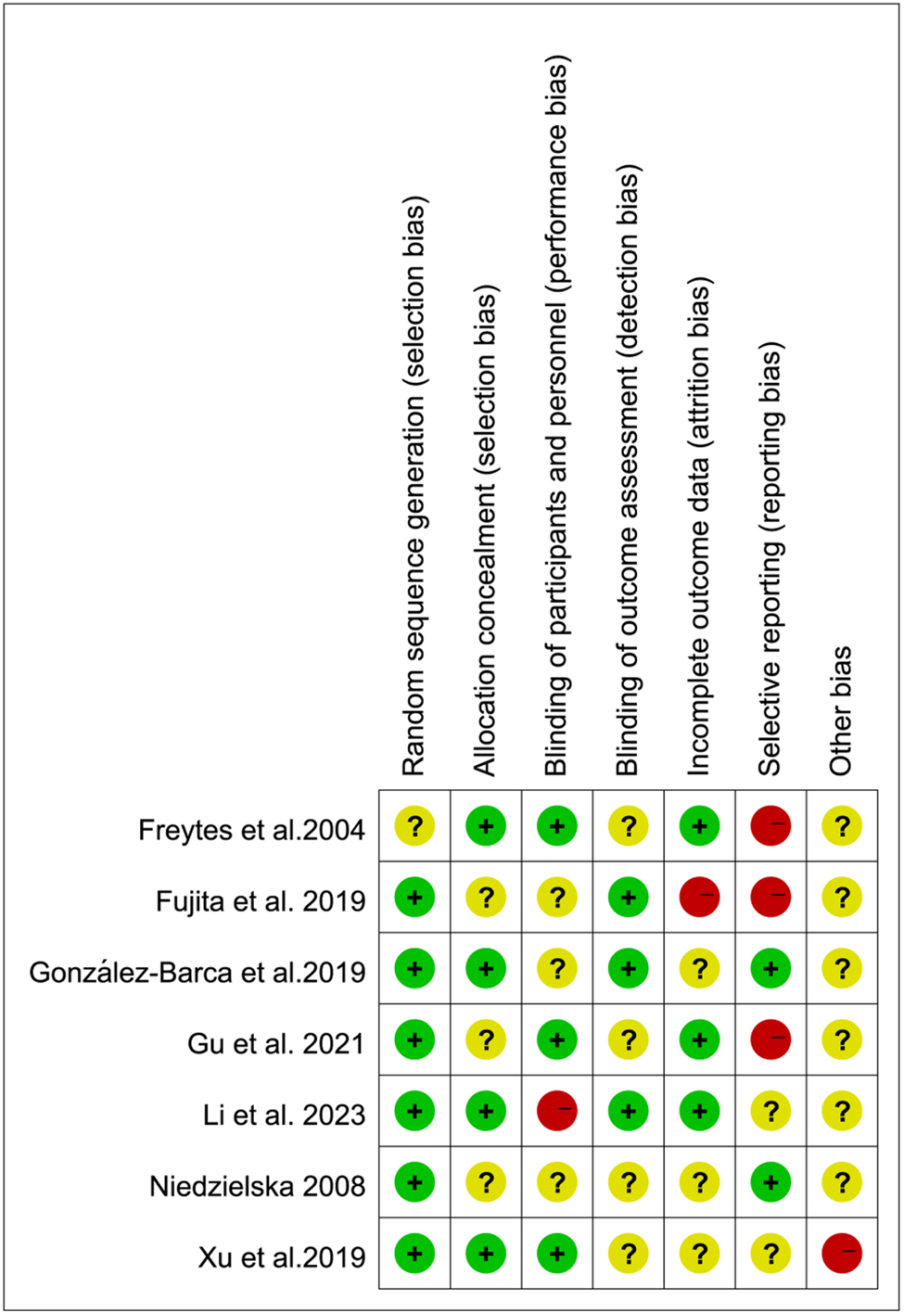
Bar chart of risk assessment of included literature.

Ultimately, seven articles were included, with a total of 972 patients. Table 1 displays the primary features of the included studies. The bias risk assessment tool provided by the Cochrane evaluation manual was used to evaluate the quality of the included literature. All the included literature reported complete test results, and their quality was rated as Grade B, indicating moderate bias, reflecting the overall quality of the literature included in this study. The evaluation results of the risk of bias are depicted in Figures 2 and 3.

**Table 1:** Baseline data of included literature

Authors	Year of publication	Country	Number of patients (allo/auto)	Treatment measures		Outcome indicators
				EXG	COG	
Gu et al. [13]	2021	China	56/72	allo-HSCT	auto-HSCT	①③
Fujita et al. [14]	2019	Japan	48/31	allo-HSCT	auto-HSCT	①②③
Li et al. [15]	2023	China	51/24	allo-HSCT	auto-HSCT	①③④⑤
Niedzielska [16]	2008	Poland	13/6	allo-HSCT	auto-HSCT	①②③
Freytes et al. [17]	2004	USA	337/164	allo-HSCT	auto-HSCT	①③
Xu et al. [18]	2019	China	7/12	allo-HSCT	auto-HSCT	③
González-Barca et al. [19]	2019	Spain	69/82	allo-HSCT	auto-HSCT	①②③

Notes: ① PFS：progression-free survival; ② AR: adverse reactions; ③ OS: overall survival; ④ CIR: cumulative incidence of relapse; ⑤ NRM: non-relapse mortality.

### 3.3 Results of meta-analysis

#### 3.3.1 OS

**Figure 4:**
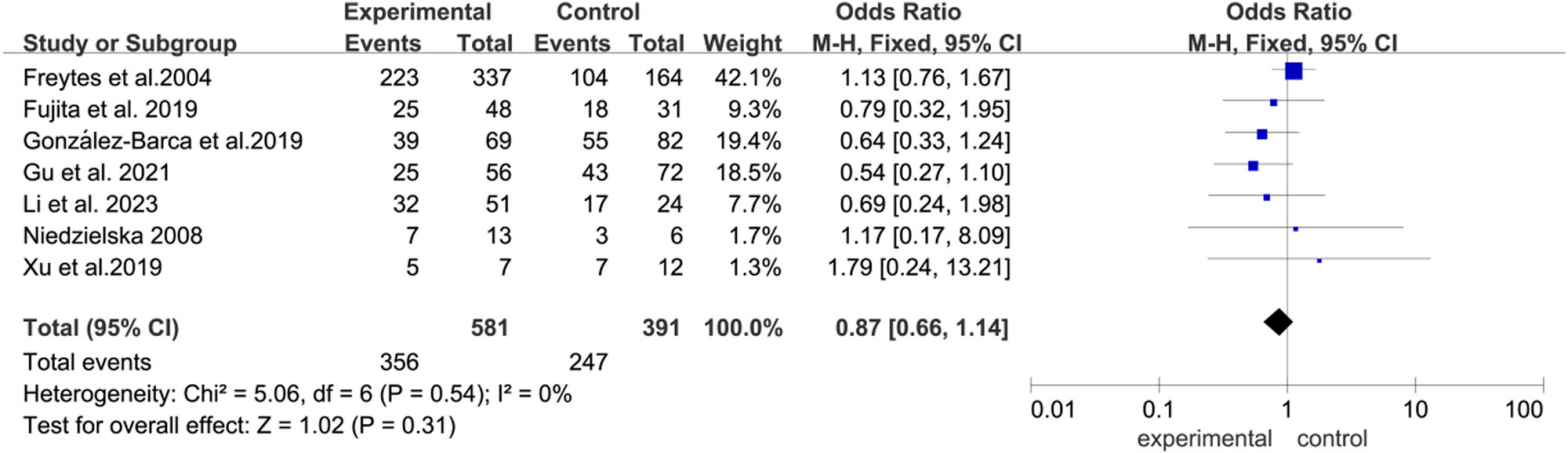
Forest plot of OS.

In this meta-analysis, all seven articles assessed OS. OS did not differ significantly (Figure 2) between allo-HSCT and auto-HSCT groups (OR  =  0.87, 95% CI: 0.66–1.14, *P*  =  0.31). Heterogeneity was not detected (*I* ^2^ = 0%) (Figure 4).

#### 3.3.2 Incidence of adverse reactions

**Figure 5:**
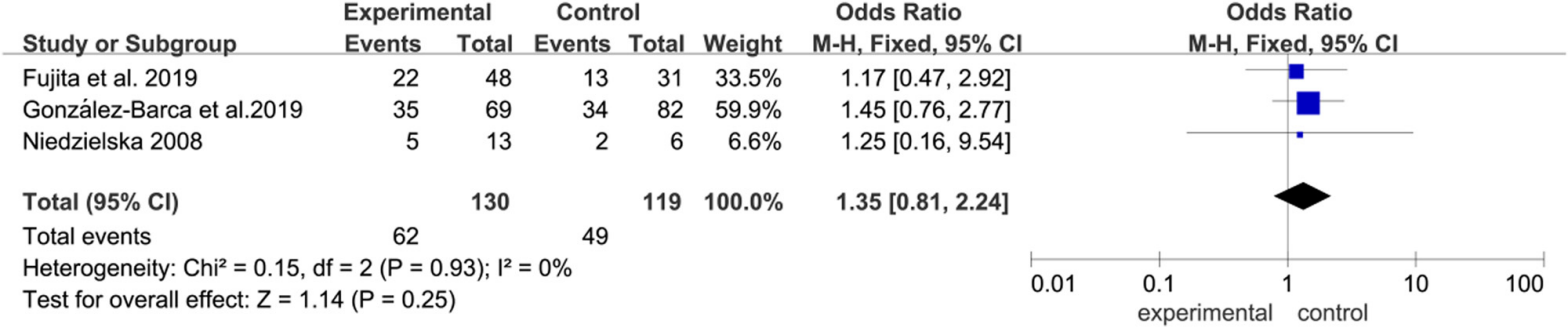
Forest plot of AR.

Three articles (Fujita et al., González-Barca et al., and Niedzielska et al.) assessed the incidence of adverse reactions. No significant difference was observed in AR (Figure 5) between allo-HSCT and auto-HSCT groups (OR  =  1.35, 95% CI: 0.81–2.24, *P*  =  0.25). Heterogeneity was not detected (*I* ^2^ = 0%).

#### 3.3.3 PFS

**Figure 6:**
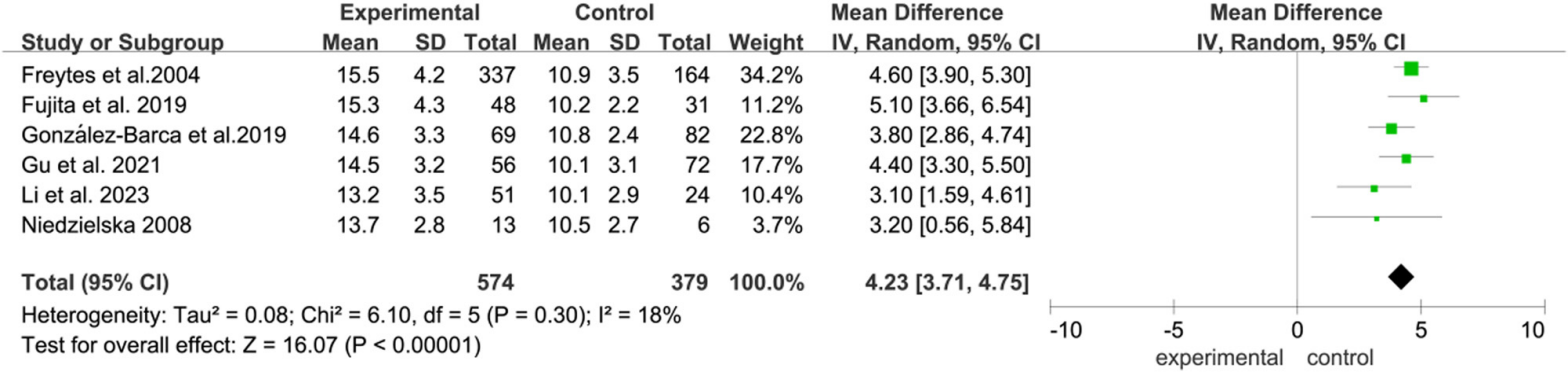
Forest plot of PFS.

Six articles (Freytes et al., Fujita et al., Gonzalez-Barca et al., Gu et al., Li et al., and Niedzielska et al.) assessed the PFS. We observed a significant difference in PFS (Figure 6) between allo-HSCT and auto-HSCT groups (OR  =  4.14, 95% CI: 2.93–5.35, *P* < 0.01). A low heterogeneity was detected (*I* ^2^ = 17%).

### 3.4 Publication bias test

**Figure 7:**
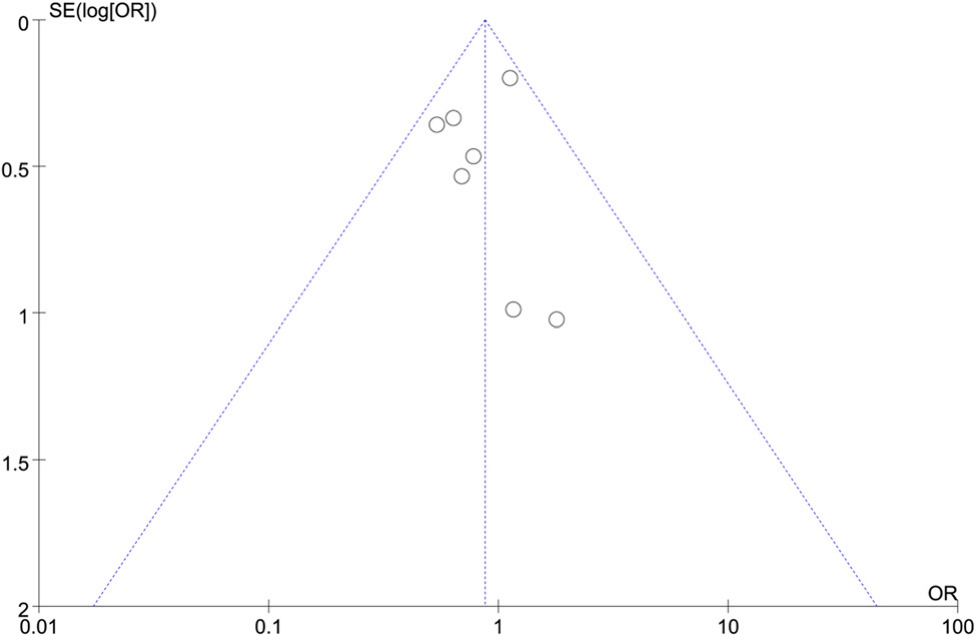
Funnel plot and Egger test of included studies.

The results of a sensitivity analysis were analyzed for their consistency. We observed no significant alterations by eliminating any one article. Publication bias was assessed using funnel plots and Egger’s linear regression, and no bias was identified (Figure 7).

## 4 Discussion

ML is a malignant tumor of lymphoid hematopoietic tissues. Patients frequently have systemic symptoms such as lymph node enlargement, skin pruritus, and fever, significantly affecting daily life and physical and mental health [20,21]. Although the incidence of this disease is not as high as that of lung cancer and digestive tract tumors, it has shown a significant upward trend in recent years [22,23]. For years, to improve the clinical outcome of patients with ML, experts have been challenging traditional treatments and developing new drugs, technologies, and methods [24]. Immunotherapy, chemoradiotherapy, and HSCT are clinically used treatments for ML. Although radiotherapy and chemotherapy have alleviated the clinical symptoms of the disease to some extent, there is still a certain gap in the expected clinical effect, and hematopoietic stem cells have brought a good prognosis to the patients [25].

Allo-HSCT, a treatment method that transplants hematopoietic stem cells from non-identical twins into patients, exerts a role in hematopoiesis or immune reconstruction and is a crucial method for the treatment of hematological diseases like hematological tumors, hematopoietic failure, or immunodeficiency [26,27]. Thanks to advances in science and technology, medical techniques, and the improvement of nursing care, the survival rate of allo-HSCT patients reaches more than 70%. With the increased quantity of allo-HSCT patients in China, the number of long-term survival patients after transplantation is enormous [28]. In the case of leukemia, the prevalence of allo-HSCT has substantially increased the survival rate of leukemia patients. The 5-year OSR increased from 19% in 1989–1994 to 37% in 2007–2012. In particular, the use of drugs such as anti-human thymic lymphocyte globulin, post-transplantation cyclophosphamide, and basiliximab and the improvement of regimens have also prominently improved the safety of allo-HSCT [29]. Auto-HSCT is more suitable for consolidation therapy after first-line induction chemotherapy for NHL with poor prognostic factors among younger patients with chemotherapy sensitivity and good physical condition or patients who are sensitive to chemotherapy after first-line treatment failure compared with allo-HSCT. Meanwhile, allo-HSCT treatment has a low recurrence rate and high disease-free survival advantages. However, donor sources have a problem; only about 30% of patients can find a compatible donor [30]. The choice of allo-HSCT or auto-HSCT is still controversial. Rauofi et al.’s study has shown that auto-HSCT and allo-HSCT achieve similar efficacy for patients with lymphoma [31]. However, there is no consensus on whether they will increase the adverse reactions of ML patients and affect the long-term survival rate of patients. The most significant advantage of meta-analysis is that the accuracy of the conclusion can be increased by expanding the sample size, which can be used to solve the inconsistency of the research results and make the conclusion more reliable compared with a single study. Therefore, this meta-analysis was based on the comparison of allo-HSCT and auto-HSCT in the treatment of ML to provide an evidence-based basis to choose the medicines for ML patients.

OS is a crucial endpoint of clinical trials on tumors and a vital indicator for evaluating the efficacy of anti-tumor drugs. Generally, the longer the OS of patients, the better the efficacy of anti-tumor treatment. A previous study found that 5-year OS after allo-HSCT (57%) was better than conventional chemotherapy and was the same as 5-year OS in automatic HSCT group [32]. In a recent prospective study [29], allo-HSCT was the first-line treatment for 29 patients with high-risk peripheral T-cell lymphoma, with a 2-year OSR of 72.5%. These studies showed that allo-HSCT and auto-HSCT are far more effective than conventional chemotherapy for these patients in relapsed or first-line treatment. This study found no significant difference in OS (OR  =  0.87, 95% CI: 0.66–1.14, *P*  =  0.31), indicating no overt difference between allo-HSCT and auto-HSCT for ML patients. Furthermore, there was no significant difference in AR between the two groups (OR  =  1.35, 95% CI: 0.81–2.24, *P*  =  0.25). We observed a significant difference in PFS between the two groups (OR  =  4.14, 95% CI: 2.93–5.35, *P* < 0.01). The reason may be that imported hematopoietic stem cells do not contain tumor cells, and there is a graft-versus tumor reaction, which may be one of the reasons why the OS of patients with allo-HSCT can be prolonged.

Due to the limited evidence currently available, this meta-analysis has the following deficiencies. (1) The included studies did not assess the relationship among OSR, OS time, and other factors (age, treatment cycle), which may cause bias in the results. (2) The quality evaluation and assessment of risk bias showed that the included literature’s quality was general, which might have methodological defects. (3) Limited by included studies, subgroup analysis of factors such as patients’ nature of work, age, and drug dose was not performed. In conclusion, there is an inevitable bias among studies, so a larger sample of clinical studies should be carried out to obtain more rigorous and convincing data.

In brief, we found that allo-HSCT offers marked PFS advantages for patients with ML. Although PFS is significantly longer among ML patients undergoing allo-HSCT compared to auto-HSCT, newly diagnosed ML patients must make a comprehensive assessment of donor availability, age, predictable quality of life, etc., before choosing initial treatment. This meta-analysis highlights the requirement for research to evaluate the two therapy approaches. The allo-HSCT appears necessary to improve ML’s negative prognostic impact. Our meta-analysis highlights the need for prospective studies to examine the role of these two treatment modalities.
